# Association of Dietary and Lifestyle Inflammation Score With Metabolic Syndrome in a Sample of Iranian Adults

**DOI:** 10.3389/fnut.2021.735174

**Published:** 2021-10-05

**Authors:** Fatemeh Dehghani Firouzabadi, Ahmad Jayedi, Elaheh Asgari, Zahra Akbarzadeh, Nasim Janbozorgi, Kurosh Djafarian, Sakineh Shab-Bidar

**Affiliations:** ^1^Department of Community Nutrition, School of Nutritional Sciences and Dietetics, Tehran University of Medical Sciences, Tehran, Iran; ^2^Department of Clinical Nutrition, School of Nutritional Sciences and Dietetics, Tehran University of Medical Sciences, Tehran, Iran

**Keywords:** diet, lifestyle, inflammation, metabolic syndrome, adults

## Abstract

**Objective:** We aimed to evaluate the association between the dietary and lifestyle inflammation score (DLIS) and metabolic syndrome (MetS) and its components in a sample of Iranian adults.

**Design:** Population-based cross-sectional study.

**Setting:** General adult population living in Tehran, Iran.

**Subjects:** We included 827 adult men and women with an age range of 18–59 years who were referred to health centers in different districts of Tehran, Iran. Dietary intake was assessed by a semiquantitative food frequency questionnaire with 168 items. The DLIS was calculated based on four components, including dietary inflammation score, physical activity, cigarette smoking, and general obesity. Higher DLIS represents a more proinflammatory diet and lifestyle. The odds ratio (OR) and 95% confidence interval (CI) of the MetS across quartiles of the DLIS was calculated by using logistic regression analysis, controlling for age, sex, energy intake, marital status, education status, and occupation.

**Results:** A total of 827 participants (31% men) were included, with a mean age of 44.7 ± 10.7 years. The prevalence of the MetS was 30.5%. The DLIS ranged between −2.35 and +3.19 (mean ± SD: 0.54 ± 1.09). There was a significant positive association between the DLIS and odds of MetS (OR _fourthvs.thefirstquartile_: 1.57, 95% CI: 1.01–2.45) in the fully adjusted model.

**Conclusion:** Our results showed a significant positive association between the DLIS and odds of MetS. The results of the present crosssectional study suggested that having a more proinflammatory lifestyle can be associated with MetS. More prospective studies are needed to confirm the findings.

## Introduction

Metabolic syndrome (MetS) consists of a set of metabolic disorders including lipid disorders, abnormal glucose homeostasis, abdominal obesity, and high blood pressure (1). All aforementioned metabolic disorders are related to an increased risk of diabetes mellitus, cardiovascular diseases (CVD), and mortality (2). The worldwide prevalence of the MetS is roughly 25% of adult populations, with increased prevalence in older age (3). In Iran, the prevalence of the MetS is 30.4%, with a considerably higher prevalence in women (35%) compared with men (26%) (4).

Several factors including genetic and environmental factors contribute to the development of the MetS (5). Among environmental factors, lifestyle and diet have a crucial role in MetS development (5, 6). According to some recent research, dietary intake of red meat, added sugar, refined carbohydrates, and iron-containing foods are related to the risk of MetS (7–9). Unhealthy lifestyle behaviors like low physical activity (<75 min/week), smoking, and a sedentary lifestyle (including watching TV or video more than 4 h/d) are also associated with the MetS (10, 11).

It is proposed that the association between the aforementioned factors and the risk of MetS may be mediated, in part, by low-grade systemic inflammation (12). There is convincing evidence that unhealthy eating behaviors, low physical activity, and cigarette smoking may induce inflammatory processes in the human body, and thereby increase the risk of MetS and other chronic diseases such as obesity (13–16).

However, previous studies have mostly evaluated the intake of a single food or nutrient, with special inflammatory potential, in association with the MetS (17, 18). In this regard, focusing on the inflammatory potential of the diet as a whole may be a better approach to reduce the potential for collinearity that may happen when considering the intake of a single food or nutrient or a certain dietary factor. In addition, the combined effect of the diet and inflammation-related lifestyle factors and their collective contribution to the development of MetS has not yet been investigated.

Recently, a new index has been developed considering all potential lifestyle-related factors that may contribute to the development of low-grade systemic inflammation (19). The dietary and lifestyle inflammation score (DLIS) accounts for five inflammation-related factors including diet, physical activity, cigarette smoking, alcohol drinking, and obesity, and thereby presents a broad picture of the effects of human diet and lifestyle on inflammatory status. Considering the underlying role of low-grade systemic inflammation in the development of chronic disease (20), focusing on the DLIS may present a better picture of the association of human lifestyle as a whole and chronic disease risk than that of a single component, such as diet or smoking. To our knowledge, no study has investigated the association of the DLIS with MetS and its components. We, therefore, aimed to investigate the potential association of the DLIS, representing patterns of lifestyle and dietary intake, in a sample of Iranian adults with MetS and its components.

## Materials and Methods

### Participants

In this crosssectional study, we recruited 850 adult men and women who were referred to health centers in five districts of Tehran, Iran from 2018 to 2019. First, a list of all health centers existing in each district was provided, and then eight health centers were randomly selected from five different districts Tehran. Next, subjects were chosen from each health center by convenience sampling method based on inclusion criteria. To obtain the number of subjects in each health center, we divided the total number of sample size (850) by the number of health centers (40).

Eligible participants who were included in the present study were apparently healthy adults who did not report any previous diagnosis of chronic diseases such as diabetes, CVD, and chronic kidney, lung, and liver diseases as indicated by a physician, who were aged between 18 and 59 years, living in Tehran city, who attended local health centers during the study period and had the willingness to take part in the study. Participants with special diets, such as weight loss or weight gain diets; adults with chronic diseases including diabetics, hormonal, and CVD; pregnant and lactating women; those receiving any special medication or supplements (slimming medicine, hormone, sedative, supplements containing thermogenic substances, such as caffeine and green tea, linoleic acid conjugate, etc.) were excluded from the study. Finally, 827 individuals were included in the analyses, after excluding subjects who had at least one incomplete variable.

### Data Collection

#### Demographic Factors

At the first visit to health centers, data about age, sex, education (having or not having university education), marriage (single or married), and occupation (employee, housekeeper, retired, unemployed) were collected by using pre-specified data extraction forms.

#### Smoking Status

We classified smoking status into two groups including no or former smoker, and current smoker.

Current smokers were those who smoked during the study. Their smoking status included low (up to five cigarettes per day), relatively low (6–15 cigarettes per day), moderate (15–25 cigarettes per day), and high (more than 25 per day). Former smokers were those who had smoked at least once in their lifetime but had not smoked in the past 30 days prior to the study. Never smokers were those who had never smoked in their lifetime.

#### Physical Activity

Physical activity was evaluated by the use of the generally validated International Physical Activity Questionnaire (IPAQ). Data were expressed as metabolic equivalent hours per week, (MET-h/week), and subjects were then grouped into two categories including no or low physical activity (<600 MET-minute/week), and moderate or high physical activity (>600 MET-minute/week) (21, 22).

#### Anthropometric and Blood Pressure Assessment and Laboratory Tests

Anthropometric variables consisting of weight, height, and waist circumference (WC) were measured. Height was measured using a wall stadiometer (Seca, Germany) and recorded to the nearest 0.1 cm. Weight was measured by digital scales for adults (808Seca, Germany with a sensitivity of 0.1 kg). WC was measured by flexible anthropometric tape midway between the iliac crest and lower rib margin with a sensitivity of 0.1 cm. Anthropometric measurements were performed without shoes and heavy clothing (coat and jacket). Body mass index (BMI) was calculated as weight in kilograms divided by the square of height in meters. Systolic (SBP) and diastolic (DBP) blood pressure were measured twice by a trained physician, by the use of a digital barometer (BC 08, Beurer, Germany) after at least 10–15 min of setting. The second measurement was performed 1–2 min later and then averaged over the two measurements. Fasting plasma glucose (FPG), serum triglyceride (TG), and high-density lipoprotein (HDL) cholesterol were measured after 10–12 h of overnight fasting from a venous blood sample. Blood samples were measured by standard methods at the Nutrition and Biochemistry Laboratory of the School of Nutritional Sciences and Dietetics at Tehran University of Medical Sciences.

### Dietary Assessment

A 168-item semiquantitative food frequency questionnaire (FFQ) was used to measure usual dietary intakes through face-to-face interviews by trained nutritionists (23). This questionnaire provided data from the food and nutrients intake of the previous year. The FFQ shows a list of food items and a standard serving size for each person. Participants selected the frequency and the portion size of their food intake for the previous year (frequency of food items includes daily, weekly, or monthly intake). Finally, the portion sizes of food consumed were converted to grams per day using “household measures."

### Calculating the DLIS

We used the method introduced by Byrd et al. to calculate the DLIS score for each participant (19). This score includes dietary inflammation score (DIS) and lifestyle inflammation score (LIS). The DIS includes a total of 19 components of diet affecting the concentrations of proinflammatory biomarkers such as interleukin- 6 (IL-6), interleukin-8 (IL-8), and C-reactive protein (CRP) or antiinflammatory biomarkers such as interleukin-10 (IL-10). Then, the inflammatory potential of each component was scored based on whether it decreases or increases circulating concentrations of pro-and antiinflammatory markers. The DIS includes leafy greens and cruciferous vegetables, tomatoes, apples and berries, deep yellow or orange vegetables and fruits, other fruits, and real fruit juices, other vegetables, legumes, fish, poultry, red and organ meats, processed meats, added sugars, high-fat dairy, low-fat dairy, coffee and tea, nuts, other fats, refined grains, starchy vegetables, and the supplement score. We used all of these components except the supplement score due to the lack of information regarding supplements used by the study participants.

The LIS includes four inflammation-related lifestyle components including cigarette smoking (“former/never” or “current”), physical activity (“high or moderate” and “low or no physical activity”), obesity as assessed by BMI (kg/m^2^) [overweight (25–29.99), or obese (≥30)], and alcohol intake. We did not include alcohol intake when calculating the DLS due to a lack of information regarding alcohol drinking in the Iranian population.

To calculate the weight of each component in the DIS and the LIS, each component was scored based on the strength of its association with an inflammation biomarker in the REGARDS case cohort study (19, 24). For this purpose, for the DIS, each dietary component was treated as a continuous variable (g/d) and then was standardized, by sex, to a mean of 0 and SD of 1.0. Multivariable linear regression was used to estimate the maximum likelihood estimates for the β coefficients, which represent the average change in the inflammation biomarker score per 1 SD increase in a dietary component. Each dietary component intake was multiplied by the weight (β coefficient) and then summed to calculate the DIS.

For the LIS, dummy variables were created for physical activity, adiposity, and smoking status, and then multivariable linear regression was applied to estimate the β coefficients to represent the average change in the inflammation biomarker score per having a certain lifestyle behavior relative to its referent category. To calculate the DLIS, the DIS and LIS were summed. A higher DLIS score (more positive) represents a more inflammatory diet and lifestyle, and a lower DLIS score (more negative) represents a less inflammatory diet and lifestyle.

### Definition of MetS and Its Components

Metabolic syndrome was defined based on National Cholesterol Education Program (NCEP ATP III) criteria (17). Presence of three or more of the following criteria was considered as MetS: (1) abdominal obesity [WC ≥ 88 cm for women and ≥ 102 cm for men]; (2) Hypertriglyceridemia [TG ≥ 150 mg/dL]; (3) low HDL concentrations [<50 mg/dL for women and <40 mg/dL for men]; (4) Hyperglycemia [FPG > 100 mg/dL]; and (5) Hypertension [SBP ≥ 130 mm Hg and/or DBP ≥ 85 mm Hg].

### Statistical Analysis

One way ANOVA test was applied to compare the means of continuous variables across quartiles of the DLIS, and chi-square test was applied to assess the frequency of categorical variables across quartiles of the DLIS. Analysis of covariance was used to compare the means of continuous variables (biochemical parameter) across quartiles of the DLIS score controlling for age, sex, marital status, occupation, and education status. The odds ratios (OR) and 95% confidence intervals (CI) of having MetS and its components and P-trend were determined through binary logistic regression in the crude and adjusted models. In the first model, we adjusted for age, sex, and energy intake. Further adjustments were made for marital status (single/married), education (under university/university graduated), and occupation (employee/housekeeper/retired/unemployed) in the second model. In all models, participants in the first quartile of the DLIS were considered as the reference group. To investigate the potential interaction of gender and the MetS component, we performed interaction analysis. All statistical analyses were performed using the Statistical Package for Social Sciences (version 26; SPSS Inc.). *P* < 0.05 was considered as a statistical significance level.

## Results

Sociodemographic characteristics and anthropometric measures of the study participants across quartiles of the DLIS are presented in Table 1. The mean age of participants was 44.7 ± 10.7 years, of whom 31.3% were men. The prevalence of MetS was 30.5%. The range of the DLIS was between −2.35 and 3.19 (mean ± SD: 0.54 ± 1.09). Compared with the subjects in the first quartile of the DLIS, those in the top quartile were at an older age and had heavier weight and larger waist circumference. The proportion of current smokers, participants with no physical activity, and patients with hypertension increased, and university-graduated participants decreased with the increase in the DLIS.

**Table 1:** Sociodemographic characteristic of the study participants across quartile of the DLIS in Tehranian adults.

**Variable**	**Q1** **(*****n*** **= 208)**		**Q2** **(*****n*** **= 208)**		**Q3** **(*****n*** **= 208)**		**Q4** **(*****n*** **= 208)**		***P*** -value*
	**Mean or N**	**SD or %**	**Mean or n**	**SD or %**	**Mean or n**	**SD or %**	**Mean or n**	**SD or %**	
DLIS	−0.35 to −0.22	−0.21 to 0.56	0.57 to 1.33	1.33 to 3.19					
Age (year)	42.6	11.4	43.3	11.1	46.6	10.0	46.3	10.0	**<0.001**
Weight (kg)	67.2	11.0	71.0	13.6	74.4	12.1	80.9	13.0	**<0.001**
WC (cm)	89.9	13.2	90.6	12.7	94.3	11.4	95.4	11.0	**<0.001**
Sex (%men)	70	34.3%	63	30.3%	66	31.7%	61	29.5%	0.73
Marital status (%married)	159	77.9%	174	83.7%	176	84.6%	158	76.3%	0.08
Current smoker (%)	10	4.9%	17	8.2%	23	11.1%	26	12.6%	**0.03**
Occupation (%)									0.62
Employee	57	27.9%	55	26.4%	51	24.5%	52	25.1%	
Housekeeper	106	52.0%	115	55.3%	121	58.5%	119	56.0%	
Retired	30	14.7%	31	14.9%	31	14.9%	31	15.0%	
Unemployed	11	5.4%	7	3.4%	5	2.4%	3	1.4%	
Obesity (BMI)									**<0.001**
Underweight/normal (<25)	122	60.4%	74	35.4%	30	13.9%	3	1.5%	
Overweight (25–29.9)	62	30.4%	99	48.6%	116	53.3%	86	41.3%	
Obese (>30)	49	9.2%	35	17.0%	62	29.8%	118	57.3%	
Physical activity (%)									0.30
No physical activity	129	63.2%	120	57.5%	139	66.8%	135	65.7%	
Moderate or heavy	75	36.8%	88	42.3%	69	33.2%	71	34.3%	
Education (%university graduated)	69	47.1%	66	31.7%	54	26.0%	68	32.9%	**<0.001**
Diabetics	103	49.8%	109	52.9%	99	47.6%	97	47.1%	0.25
Patients with hypertension	52	25.2%	67	32.5%	83	39.9%	83	39.9%	**0.003**

*BMI, body mass index; DLIS, dietary and lifestyle inflammation score; Q, quartile; WC, waist circumference*.

*The Values are mean ± SD for continuous variables and percent for categorical variables*.

* *p-values resulted from the analysis of one-way ANOVA for continuous variables and chi-square test for categorical variables. Statistically significant results are provided in bold values*.

The values of biochemical parameters of the study participants across quartiles of the DLIS are indicated in Table 2. Participants in the top quartile of the DLIS tend to have higher levels of SBP, DBP, and FPG, but the changes across quartiles were not significant. The circulating concentrations of HDL and TG did not change with the increase in the DLIS. Stratified analysis by sex indicated that values of cardiometabolic risk factors did not change significantly across quartiles of the DLIS either in men or in women (Supplementary Tables 1, 2). To test for potential effect modification by energy intake, we investigated the associations in participants with lower and higher median energy intake. The results suggested no effect modification by energy intake, except for SBP for which there was a significant trend toward higher SBP along with the increase in the DLIS (Supplementary Tables 3, 4).

**Table 2:** Cardiometabolic profile of the study participants across quartile of the DLIS in Tehranian adults.

**Variables**	**Q1 (*****n*** **= 208)**		**Q2 (*****n*** **= 208)**		**Q3 (*****n*** **= 208)**		**Q4 (*****n*** **= 208)**		***P*** -value*
	**Mean**	**SD**	**Mean**	**SD**	**Mean**	**SD**	**Mean**	**SD**	
SBP (mm Hg)	115.7	23.0	119.2	23.9	122.4	22.3	121.6	19.5	0.12
DBP (mm Hg)	76.1	13.1	77.7	14.4	78.7	12.2	80.0	13.6	0.18
FBS (mg/dL)	104.6	22.3	109.1	33.7	106.6	40.1	110.1	62.4	0.57
TG (mg/dL)	146.2	77.2	144.5	79.8	146.2	80.7	146.1	81.9	0.98
HDL (mg/dL)	50.4	10.2	49.3	9.9	50.1	10.4	49.4	9.9	0.58

*FPG, fasting plasma glucose; DBP, diastolic blood pressure; DLIS, dietary and lifestyle inflammation score; HDL, high-density lipoprotein; Q, quartile; SBP, systolic blood pressure; TG, triglyceride*.

*The Values are based on mean ± SD*.

* *p-value is obtained by ANCOVA and adjusted for age, sex, education status, occupation status, and marital status, and energy intake*.

Dietary intakes of the study participants across quartiles of the DLIS are reported in Table 3. The intakes of energy and macronutrients did not differ significantly across quartiles. Participants in the highest quartile of the DLIS had lower intakes of leafy greens and cruciferous vegetables, tomatoes, apples and berries, deep yellow or orange vegetables and fruit, other fruits and real fruit juices, other vegetables, legumes, fish, poultry, red and organ meats, high-fat dairy, low-fat dairy, coffee and tea, nuts and other fats and higher intakes of added sugars, processed meats, refined grains, and starchy vegetables.

**Table 3:** Dietary intakes of the study participants across quartile of the DLIS in Tehranian adults.

**Variables**	**Q1 (*****n*** **= 208)**		**Q2 (*****n*** **= 208)**		**Q3 (*****n*** **= 208)**		**Q4 (*****n*** **= 208)**		***P*** -value*
	**Mean**	**SD**	**Mean**	**SD**	**Mean**	**SD**	**Mean**	**SD**	
Energy (kcal/d)	2534	1364	2483	1093	2724	3450	2535	1084	0.69
Carbohydrate (g/d)	370	211	369	173	427	827	373	170	0.64
Protein (g/d)	84.8	41.5	86.8	46.1	89.0	64.2	85.4	37.4	0.78
Total fat (g/d)	85.8	65.0	79.2	46.3	81.5	48.8	82.7	43.5	0.65
Fiber (g/d)	27.6	12.5	17.8	10.1	28.4	12.5	20.1	11.8	0.66
PUFA (g/d)	18.0	17.0	16.1	9.35	16.9	11.5	17.5	12.5	0.88
MUFA (g/d)	29.2	48.8	24.2	20.4	24.8	14.7	25.1	14.5	0.19
Leafy greens and cruciferous vegetables (g/d)	36.2	28.7	31.2	26.3	31.0	27.6	25.7	21.5	**<0.001**
Tomatoes (g/d)	39.5	49.4	28.1	27.6	20.7	25.7	18.5	17.3	**<0.001**
Apples and berries (g/d)	36.8	5.7	41.6	81.3	22.2	53.8	18.8	32.2	**<0.001**
Deep yellow or orange vegetables and fruit (g/d)	78.7	93.1	54.5	63.0	34.5	40.7	22.3		**<0.001**
Other fruits and real fruit juices (g/d)	378	352	279	32.5	229	351	160	130	**<0.001**
Other vegetables (g/d)	44.7	36.3	67.6	29.7	51.4	22.3	16.1	20.4	**<0.001**
Legumes (g/d)	77.9	10.5	61.5	95.2	50.1	42.1	46.1	35.6	**<0.001**
Fish (g/d)	1.37	9.24	0.17	0.69	0.34	2.12	0.32	1.36	**0.04**
Poultry (g/d)	44.1	78.4	37.4	76.3	31.2	73.3	18.0	55.0	**<0.001**
Red and organ meats (g/d)	299	439	291	392	235	276	201	187	**0.001**
Processed meats	18.3	16.3	22.0	18.3	22.2	35.6	29.4	27.4	**<0.001**
Added sugars (g/d)	598	499	643	601	661	419	912	1302	**<0.001**
High-fat dairy (g/d)	321	273	254	252	252	312	196	301	**<0.001**
Low-fat dairy (g/d)	20.2	28.9	19.9	15.2	15.2	16.8	14.8	22.9	**0.007**
Coffee and tea (g/d)	8.06	24.0	6.21	3.99	3.99	8.53	4.36	19.8	**0.018**
Nuts (g/d)	23.5	26.3	21.0	14.0	14.0	29.8	12.5	30.6	**<0.001**
Other fats (g/d)	28.3	44.1	19.8	17.7	17.7	32.4	16.9	25.6	**0.001**
Refined grains and starchy vegetables (g/d)	472	376	484	487	487	318	555	318	**0.013**

*DLIS, dietary and lifestyle inflammation score; MUFA, monounsaturated fat; Q, quartile; PUFA, polyunsaturated fat*.

*Data are mean ± SD*.

* *P for the trend is from ANOVA (One-way ANOVA). Statistically significant results are provided in bold values*.

Odds ratios and 95% CI of the MetS and its component across quartiles of the DLIS are shown in Table 4. In the crude model, there was a significant positive association between the DLIS and odds of MetS (OR_fourthvs.firstquartile_: 1.69, 95% CI: 1.10, 2.60, *P* trend: 0.01). The significant positive association persisted in the fully adjusted model that controlled for age, sex, energy intake, marital status, occupation, and education status (OR _fourthvs.firstquartile_: 1.57, 95% CI: 1.01, 2.45, *P* trend: 0.03). In addition, there was a significant positive association between the DLIS and increased likelihood of having central obesity (OR_fourthvs.firstquartile_: 3.45, 95% CI: 2.18, 5.46, *P* trend: <0.001) and hypertension (OR_fourthvs.firstquartile_: 1.75, 95% CI: 1.13, 2.70, *P* trend: 0.03) after controlling for confounders. There was no significant association between other components of MetS and the DLIS, either before or after controlling for confounding variables. Table 4 also indicates the interaction of gender with Mets and its components according to DLIS. The analyses indicated that there was a significant interaction between MetS and its components, except for hyperglycemia and hypertriglyceridemia.

**Table 4:** Association between the DLIS and MetS and its component in Tehranian adults (Odd ratios and 95% CI).

**Variable**	**Q1** **(*****n*** **= 208)**	**Q2** **(*****n*** **= 208)**	**Q3** **(*****n*** **= 208)**	**Q4** **(*****n*** **= 208)**	***P*** -trend*	***P*** ** for interaction with gender**
**MetS (cases, n)**	49	60	70	71		**<0.001**
Crude	1.0	1.32 (0.85–2.05)	1.63 (1.06–2.51)	1.69 (1.10–2.60)	**0.01**	
Model 1	1.0	1.28 (0.91–1.94)	1.55 (1.00–2.42)	1.60 (1.03–2.49)	**0.02**	
Model 2	1.0	1.26 (0.81–1.98)	1.55 (0.99–2.42)	1.57 (1.01–2.45)	**0.03**	
**Low HDL**						**<0.001**
Crude	1.0	1.25 (0.84–1.86)	1.14 (0.77–1.69)	1.13 (0.91–2.01)	0.19	
Model 1	1.0	1.21 (0.79–1.84)	1.14 (0.75–1.75)	1.33 (0.87–2.04)	0.22	
Model 2		1.20 (0.79–18.3)	1.15 (0.75–1.76)	1.33 (0.87–2.04)	0.22	
**Central obesity**						**<0.001**
Crude	1.0	1.78(1.19–2.66)	2.39 (1.60–3.56)	3.65 (2.43–5.48)	**<0.001**	
Model 1	1.0	1.83 (1.18–2.83)	2.04 (1.31–3.17)	3.40 (2.19–5.40)	**<0.001**	
Model 2	1.0	1.79 (1.15–2.78)	2.00 (1.58–3.12)	3.45 (2.18–5.46)	**<0.001**	
**Hyperglycemia**						0.29
Crude	1.0	1.13 (0.77–1.66)	0.91 (0.62–1.34)	0.89 (0.61–1.32)	0.39	
Model 1	1.0	1.13 (0.77–1.67)	0.93 (0.63–1.37)	0.90 (0.61–1.34)	0.44	
Model 2	1.0	1.14 (0.77–1.68)	0.94 (0.63–1.39)	0.90 (0.61–1.33)	0.43	
**Hypertriglyceridemia**						0.45
Crude	1.0	1.02 (0.69–1.53)	0.97 (0.65–1.44)	1.0 (0.67–1.50)	0.95	
Model 1	1.0	1.04 (0.70–1.55)	1.0 (0.67–1.50)	1.05 (0.70–1.57)	0.86	
Model 2	1.0	1.03 (0.69–1.54)	1.0 (0.67–1.50)	1.05 (0.70–1.57)	0.85	
**Hypertension**						**<0.001**
Crude	1.0	1.31 (0.86–2.00)	1.88 (1.24–2.83)	1.99 (1.32–3.00)	**0.04**	
Model 1	1.0	1.26 (0.81,1.95)	1.63 (1.06,2.51)	1.72 (1.12,2.65)	**0.04**	
Model 2	1.0	1.23 (0.79,1.91)	1.64 (1.07,2.53)	1.75 (1.13,2.70)	**0.03**	

*DLIS, dietary and lifestyle inflammation score; HDL, high-density lipoprotein cholesterol; MetS, metabolic syndrome; Q, quartile*.

*Data are presented as odds ratio (95% CI)*.

* *P-trend is obtained by logistic regression analysis. Statistically significant results are provided in bold values*.

*Model 1: adjusted for age, sex, and energy intake*.

*Model 2: additionally adjusted for marital status, education status, and occupation*.

The association between DLIS and MetS and its components across either sex is presented in Supplementary Tables 5, 6. In men, higher DLIS was not associated with the MetS and its components, except for low HDL concentration (OR_fourthvsfirstquartile_: 3.36, 95% CI: 1.17, 9.63, *P* trend: 0.05) (Supplementary Table 5). In women, fourth compared with the first quartile of the DLIS was associated with a higher odds of MetS (OR: 1.86, 95% CI: 1.12, 3.07), central obesity (OR: 5.33, 95% CI: 2.99, 9.49), and hypertension (OR: 2.30, 95% CI: 1.26, 4.22) (Supplementary Table 6). To test for potential effect modification by calorie intake, we tested the associations in individuals with lower and higher median energy intake. The results suggested that higher DLIS was associated with a higher likelihood of having MetS in those who consumed lower median energy intake (OR_ <2241kcal/d_: 2.06, 95% CI: 1.10, 3.87), but not in those with higher median energy intake (Supplementary Tables 7, 8).

The ORs and 95% CI of the MetS and its component across quartiles of the DIS and LIS are presented in Supplementary Tables 5, 6, respectively. There was no association between DIS and MetS and its components either in the crude or in the fully adjusted models. However, top versus bottom quartile of the LIS was significantly associated with an increased likelihood of MetS (OR: 2.40, 95% CI: 1.54, 3.81), hyperglycemia (1.52, 95% CI: 1.01, 2.29), hypertension (OR: 1.85, 95% CI: 1.13, 3.01), and central adiposity (OR: 10.2, 95% CI: 6.12, 17.10) (Supplementary Tables 9, 10). To determine whether the associations could have been affected by BMI, we recalculated the LIS after the exclusion of BMI. The results indicated that higher LIS, without BMI, was still associated with a higher odds of having MetS (OR_secondvs.firsttertile_: 1.73, 95% CI: 1.25, 2.04), hyperglycemia (OR_thirdvs.firsttertile_: 2.29, 95% CI: 1.37, 3.84), and central adiposity (OR_thirdvs.firsttertile_: 1.90, 95% CI: 1.05, 3.41) (Supplementary Table 11).

## Discussion

In this crosssectional study, we found that higher adherence to a proinflammatory diet and lifestyle had a significant positive association with odds of MetS. Also, there was a significant positive association between the DLIS with abdominal obesity and hypertension. No significant association was found regarding other components of MetS. To the best of our information, this is the first study that assessed the association of DLISs with MetS and its components.

In this study, participants with a proinflammatory diet and lifestyle were more likely to have MetS. An international panel recommendation suggested that some lifestyle modifications such as smoking cessation, being more physically active, and adopting a healthy diet can reduce the risk of developing MetS (5). A prospective study showed more proinflammatory diets and lifestyles can be associated with higher all-cause, all-cancer, and all-CVD mortality risks among women (25). Our findings regarding the positive association of the DLIS and the MetS are consistent with those of previous investigations regarding the association of the components of the DLIS and the risk of MetS.

Several crosssectional investigations have found a negative association between physical activity and the likelihood of having MetS (26–29). Cigarette smoking has also been shown to be an independent risk factor of MetS (30–32). In addition, overweight and obesity are the main underlying causes of developing MetS in both children and adults (33, 34). According to our findings, the three components of LIS including obesity, low physical activity, and smoking, strongly play a role in increased systemic inflammation in the body. On the other hand, according to the article of Byrd et al. (19), these three components make up a large share of the weights assigned to each of the DLIS components. Due to the high frequency of overweight and obese people and people with low physical activity in the study population, the results are not far from expectations.

There is also evidence that dietary patterns with high inflammatory potential can be associated with MetS. It has been shown that dietary patterns with high intake of proinflammatory components such as refined starches, sugar, and saturated and trans fatty acids, and poor intake of antiinflammatory dietary components such as dietary antioxidants, fruits, vegetables, and whole grains may increase levels of inflammatory biomarkers and as a result, can increase the risk of MetS and coronary heart diseases (35). A population-based prospective cohort study in Iran found a strong positive association between empirical dietary inflammatory pattern score and the risk of developing MetS (36).

Two crosssectional research on Iranian and Korean adults also showed a significant association between dietary inflammatory index (DII), as being representative of the inflammatory potential of the diet, and odds of MetS (37, 38). In contrast, Ghorabi et al. found no significant association between DII and risk of MetS (39). A recent metaanalysis of crosssectional studies also suggested a nonsignificant positive association between DII and the risk of MetS (40). However, DII has a heavy focus on nutrients (compared with food groups in the present study). Variations in sample size and study design, different parameters used for describing lifestyle and diet, different tools used for dietary and lifestyle assessment, and different criteria used for the definition of MetS have all led to various results.

When we measured components of MetS, the results indicated that following a diet and lifestyle with high inflammatory potential was positively associated with increased waist circumference and hypertension. No other significant association was observed between the DLIS and other components of the MetS like lipid profiles. In line with our results, a prospective cohort study in Spain indicated that proinflammatory lifestyle features such as smoking, low physical activity, and heavy drinking may have a significant association with increased waist circumference and high blood pressure (41). A longitudinal study on Australians showed that adherence to a proinflammatory diet was associated with an increase in hypertension during a period of 12 years (42). Moreover, several studies reported that a proinflammatory diet may be significantly associated with increased waist circumference and central obesity (36, 43). In line with our findings, a cross-sectional study on Indonesian adults indicated that a more proinflammatory diet did not relate to lipid profiles such as triglyceride and high-density lipoprotein (HDL) (44). A crosssectional study determined that overweight/obese female adolescents with low physical activity presented higher values of anthropometric indices, blood pressure, and hs-CRP levels, and lower HDL concentrations (45). Moreover, an observational study showed that cigarette smoking was related to dyslipidemia among Iraqi smokers (46). In contrast to our results, Abdurahman et al. found no significant association between a proinflammatory diet with increased waist circumference and high blood pressure (47). More research is needed to investigate the association of the DLIS and MetS and its components.

In this regard, the source of inflammation during obesity is not yet completely unknown (48). The inflammation could be detected by adiposity, but this association could be bidirectional and a vicious cycle could produce positive feedback (49). In addition, having a proinflammatory lifestyle and eating certain foods that have been associated with inflammation may intensify the effects of adiposity (50–52). Based on recent studies, there is an association between diet and lifestyle components with inflammatory biomarkers (19). Several studies have shown an association between the lower serum levels of proinflammatory biomarkers such as CRP or TNF-α and a higher intake of fruits and vegetables, fish, legumes, and nuts (35, 51–54). Also, several studies have shown that proinflammatory lifestyle features, such as low physical activity, cigarette smoking, and increased BMI had a positive association with increased levels of inflammatory biomarkers (55, 56). The aforementioned investigations have mainly focused on one of the inflammation-related lifestyle components and synergistic effects of different inflammation-related factors, and their relative contribution in inflammatory status has not been considered. In the present study, we used a new-developed innovative index that accounts for all important inflammation-related lifestyle components and considers their collative contribution to low-grade systemic inflammation. Our results showed a relatively strong positive relationship between having a more proinflammatory lifestyle with the odds of MetS, central obesity, and hypertension.

The strength of this study is that all data collection was conducted by skilled nutritionists by using valid and reliable questionnaires. Also, we used the DLIS as exposure which incorporates influences of both diet and lifestyle to systemic inflammation, and this was validated by a previous study. In addition, we used a food-based dietary index to present the inflammatory potential of the diet that facilitates its use in clinical and public health programs. However, the study has some limitations. Due to the crosssectional design of this study, the temporal sequence and as a result, causation cannot be proved. Therefore, prospective studies are needed to confirm the findings. Due to the relatively small sample size and larger population of women than men, we may not be able to generalize these results to other populations. Also, FFQ relies on memory and this can be one of the limitations of reading. Moreover, in this study data on 21 components were available for the development of the DLIS, and two items including supplement use and alcohol drinking were not included in the score that might affect our results. In addition, since BMI is a component of the LIS, the results of abdominal obesity should be interpreted with caution. However, we repeated the analyses after exclusion of BMI which indicated that higher LIS, without BMI, was still associated with MetS and central obesity. Despite control for several confounding variables in our study, the potential effects of remaining confounders should be considered. In addition, the main components of the LIS such as physical activity are associated with adiposity. The main components of the DIS including sugars, fats, refined grains, red meat, and vegetables have also obesogenic properties and thus, a diet with a higher inflammation score has higher obesogenic properties. In fact, the diet inflammation score is reflective of an obesogenic diet. Thus, both the dietary and lifestyle components of the score are reflective of exposure to a pro-obesity set of choices. This may suggest that the associations found in the present study may be due to obesogenic properties of the score rather than inflammatory properties of the score.

## Conclusions

In conclusion, we found a significant positive association between the DLIS and odds of MetS. The results showed that adopting a more proinflammatory lifestyle may increase the risk of MetS. However, the associations found in the present study may be due to obesogenic properties of the components of the score and not due to their inflammatory properties. Further researches, specifically those with prospective nature, are required to approve the present results.

## Data Availability Statement

The original contributions presented in the study are included in the article Supplementary Material, further inquiries can be directed to the corresponding author.

## Ethics Statement

This study was conducted according to the guidelines laid down in the Declaration of Helsinki and all procedures involving research study participants were approved by the Ethics Committee of Tehran University of Medical Sciences (Ethics Number: IR.TUMS.VCR.REC.1397.157). Written informed consent was obtained from all subjects/patients. The patients/participants provided their written informed consent to participate in this study.

## Author Contributions

SS-B and KD contributed to the conception/design of the research. ZA and NJ contributed to the acquisition of data. FD and EA participated in the analysis and interpretation of the data. FD drafted the manuscript. AJ critically revised the manuscript. SS-B agrees to be fully accountable for ensuring the integrity and accuracy of the work. All authors read and approved the final manuscript.

## Funding

This manuscript has been granted by the Tehran University of Medical Sciences (Grant No: 40186). The funder had no role in the design, analysis, or writing of this article.

## Conflict of Interest

The authors declare that the research was conducted in the absence of any commercial or financial relationships that could be construed as a potential conflict of interest.

## Publisher's Note

All claims expressed in this article are solely those of the authors and do not necessarily represent those of their affiliated organizations, or those of the publisher, the editors and the reviewers. Any product that may be evaluated in this article, or claim that may be made by its manufacturer, is not guaranteed or endorsed by the publisher.
