# Supplementary material for: Association of Dietary and Lifestyle Inflammation Score With Metabolic Syndrome in a Sample of Iranian Adults
Source: Front Nutr. 2021 Oct 5;8:735174. doi: 10.3389/fnut.2021.735174 (PMC8523681; doi:10.3389/fnut.2021.735174)
Supplement: Supplementary file 1 [file Data_Sheet_1.pdf]

Supplementary Materials including Supplementary Tables 1–12

Supplementary Table 1. Cardiometabolic profile of the study participants across quartiles of the dietary and lifestyle inflammation score in men.

Variables	Q1 (n=65)		Q2 (n=65)		Q3 (n=65)		Q4 (n=65)		P-value*
	Mean	SD	Mean	SD	Mean	SD	Mean	SD	
SBP (mm Hg)	119	26.0	119	26.9	123	18.8	122	17.7	0.34
DBP (mm Hg)	77.4	12.8	78.4	18.3	79.6	14.5	80.3	9.79	0.51
FBS (mg/dL)	104	17.1	106	38.3	112	37.8	119	58.3	0.34
TG (mg/dL)	146	76.7	144	64.5	143	75.6	162	89.8	0.48
HDL (mg/dL)	50.6	10.0	48.0	9.42	49.3	10.0	48.8	10.8	0.45

Abbreviations: FPG, fasting plasma glucose; DBP, diastolic blood pressure; DLIS, dietary and lifestyle inflammation score; HDL, high-density lipoprotein; Q, quartile; SBP, systolic blood pressure; SD, standard deviation; TG, triglyceride.

*P-value is Obtained by ANCOVA and adjusted for age, education status, Occupation status, marital status, and energy intake.

Supplementary Table 2. Cardiometabolic profile of the study participants across quartile of the dietary and lifestyle inflammation score in women.

Variables	Q1 (n=142)		Q2 (n=140)		Q3 (n=143)		Q4 (n=142)		P-value*
	Mean	SD	Mean	SD	Mean	SD	Mean	SD	
SBP (mm Hg)	113	21.2	119	22.7	121	24.8	122	18.9	0.14
DBP (mm Hg)	75.6	13.1	77.4	12.5	77.8	12.3	80.4	13.9	0.22
FBS (mg/dL)	105	24.6	109	37.6	108	41.1	105	29.5	0.66
TG (mg/dL)	145	78.1	144	85.2	149	84.7	139	75.9	0.64
HDL (mg/dL)	50.3	10.2	50.2	10.3	50.6	10.6	49.8	9.50	0.90

Abbreviations: FPG, fasting plasma glucose; DBP, diastolic blood pressure; DLIS, dietary and lifestyle inflammation score; HDL, high-density lipoprotein; Q, quartile; SBP, systolic blood pressure; SD, standard deviation; TG, triglyceride.

*P-value is Obtained by ANCOVA and adjusted for age, education status, Occupation status, marital status, and energy intake.

Supplementary Table 3. Cardiometabolic profile of the study participants across quartile of the dietary and lifestyle inflammation score in participant who intake ≤2241 kcal of energy per day.

Variables	Q1 (n=107)		Q2 (n=108)		Q3 (n=102)		Q4 (n=96)		P-value*
	Mean	SD	Mean	SD	Mean	SD	Mean	SD	
SBP (mm Hg)	116	22.4	117	28.4	119	26.3	121	20.6	0.73
DBP (mm Hg)	76.8	12.5	76.6	16.7	77.5	12.2	80.6	14.8	0.29
FBS (mg/dL)	105	25.0	107	33.7	106	39.4	106	32.1	0.98
TG (mg/dL)	149	79.6	147	86.1	151	86.0	131	60.7	0.34
HDL (mg/dL)	50.5	9.79	48.9	9.88	50.1	10.4	51.7	9.42	0.31

Abbreviations: FPG, fasting plasma glucose; DBP, diastolic blood pressure; DLIS, dietary and lifestyle inflammation score; HDL, high-density lipoprotein; Q, quartile; SBP, systolic blood pressure; SD, standard deviation; TG, triglyceride.

*P-value is Obtained by ANCOVA and adjusted for age, sex, education status, Occupation status, and marital status.

Supplementary Table 4. Cardiometabolic profile of the study participants across quartile of the dietary and lifestyle inflammation score in participant who intake >2241 kcal of energy per day.

Variables	Q1 (n=100)		Q2 (n=97)		Q3 (n=106)		Q4 (n=111)		P-value*
	Mean	SD	Mean	SD	Mean	SD	Mean	SD	
SBP (mm Hg)	114	23.5	120	18.1	123	19.4	122	16.6	**0.04**
DBP (mm Hg)	75.4	13.5	79.0	11.7	79.2	13.8	80.2	10.8	0.14
FBS (mg/dL)	104	19.6	109	41.8	113	40.5	112	79.7	0.72
TG (mg/dL)	142	75.4	140	70.8	144	77.9	156	94.1	0.50
HDL (mg/dL)	50.2	10.5	50.2	10.2	49.5	10.5	47.5	9.99	0.18

Abbreviations: FPG, fasting plasma glucose; DBP, diastolic blood pressure; DLIS, dietary and lifestyle inflammation score; HDL, high-density lipoprotein; Q, quartile; SBP, systolic blood pressure; SD, standard deviation; TG, triglyceride.

*P-value is Obtained by ANCOVA and adjusted for age, sex, education status, Occupation status, and marital status.

Supplementary Table 5. Association between the dietary and lifestyle inflammation score and metabolic syndrome in men (Odd ratios and 95% confidence intervals).

**Variable**	**Q1(n=65)**	**Q2(n=65)**	**Q3(n=65)**	**Q4(n=65)**	**P-trend***
**MetS** (cases, n)	13	14	14	15	
Crude	1.0	1.07(0.46, 2.51)	1.07(0.46, 2.51)	1.17(0.50, 2.72)	0.71
Model 1	1.0	1.02(0.43, 2.40)	1.04(0.44, 2.45)	1.16(0.50, 2.70)	0.72
Model 2	1.0	1.03(0.42, 2.53)	1.09(0.45, 2.65)	1.27(0.53, 3.02)	0.57
**Low HDL**					
Crude	1.0	2.45(0.87, 6.93)	1.78(0.60, 5.24)	2.95(1.06, 8.17)	0.07
Model 1	1.0	2.51(0.88, 7.15)	1.82(0.61, 5.36)	2.94(1.06, 8.15)	0.08
Model 2	1.0	2.93(0.98, 8.75)	2.14(0.69, 6.60)	3.36(1.17, 9.63)	**0.05**
**Central obesity**					
Crude	1.0	1.56(0.73, 3.36)	1.26(0.58, 2.75)	1.17(0.53, 2.56)	0.85
Model 1	1.0	1.47(0.67, 3.20)	1.22(0.55, 2.70)	1.16(0.52, 2.58)	0.84
Model 2	1.0	1.52(0.68, 3.42)	1.20(0.53, 2.72)	1.25(0.55, 2.83)	0.75
**Hyperglycemia**					
Crude	1.0	0.94(0.47, 1.87)	1.28(0.64, 2.54)	1.06(0.53, 2.11)	0.65
Model 1	1.0	0.97(0.48, 1.95)	1.30(0.65, 2.62)	1.08(0.54, 2.18)	0.62
Model 2	1.0	0.95(0.46, 1.95)	1.28(0.62, 2.60)	1.10(0.54, 2.22)	0.60
**Hypertriglyceridemia**					
Crude	1.0	0.87(0.43, 1.78)	0.66(0.32, 1.37)	1.54(0.77, 3.09)	0.33
Model 1	1.0	0.92(0.96, 1.01)	0.68(0.33, 1.42)	1.55(0.77, 3.11)	0.34
Model 2	1.0	0.88(0.42, 1.85)	0.69(0.33, 1.46)	1.68(0.82, 3.42)	0.23
**Hypertension**					
Crude	1.0	1.21(0.57, 2.58)	1.28(0.60, 2.73)	0.90(0.42, 1.96)	0.83
Model 1	1.0	1.01(0.45, 2.27)	1.18(0.5, 2.66)	0.92(0.40, 2.10)	0.94
Model 2	1.0	0.98(0.41, 2.33)	1.21(0.51, 2.88)	0.99(0.42, 2.34)	0.89

Abbreviations: LIS: lifestyle inflammation score; HDL, high-density lipoprotein cholesterol; MetS: metabolic syndrome, Q, quartile.

Data are presented as odds ratio (95% CI).

*P-trend is obtained by logistic regression analysis.

Model 1: adjusted for age and energy intake.

Model 2: additionally adjusted for marital status, education status, and occupation.

Supplementary Table 6. Association between the dietary and lifestyle inflammation score and metabolic syndrome in women (Odd ratios and 95% confidence intervals).

**Variable**	**Q1(n=142)**	**Q2(n=140)**	**Q3(n=143)**	**Q4(n=142)**	**P-trend***
**MetS** (cases, n)	44	55	75	72	
Crude	1.0	1.44(0.88, 2.35)	2.45(1.51, 3.98)	2.29(1.41, 3.71)	**<0.001**
Model 1	1.0	1.43(0.86, 2.36)	2.06(1.25, 3.40)	1.85(1.12, 3.06)	**0.006**
Model 2	1.0	1.40(0.84, 2.33)	2.08(1.26, 3.44)	1.86(1.12, 3.07)	**0.006**
**Low HDL**					
Crude	1.0	1.15(0.72, 1.84)	1.10(0.69, 1.75)	1.15(0.72, 1.83)	0.61
Model 1	1.0	1.15(0.72, 1.85)	1.14(0.71, 1.83)	1.18(0.73, 1.91)	0.51
Model 2	1.0	1.16(0.72, 1.86)	1.14(0.71, 1.83)	1.16(0.72, 1.88)	0.55
**Central obesity**					
Crude	1.0	1.83(1.13, 2.94)	3.21(1.98, 5.22)	6.46(3.81, 10.9)	**<0.001**
Model 1	1.0	1.97(1.17, 3.33)	2.58(1.52, 4.38)	5.27(2.98, 9.32)	**<0.001**
Model 2	1.0	1.93(1.14, 3.26)	2.57(1.51, 4.38)	5.33(2.99, 9.49)	**<0.001**
**Hyperglycemia**					
Crude	1.0	1.00(0.62, 1.59)	0.76(0.48, 1.22)	0.75(0.47, 1.20)	0.13
Model 1	1.0	1.00(0.62, 1.59)	0.75(0.47, 1.21)	0.74(0.46, 1.19)	0.13
Model 2	1.0	0.98(0.61, 1.58)	0.75(0.47, 1.22)	0.74(0.46, 1.20)	0.15
**Hypertriglyceridemia**					
Crude	1.0	1.12(0.69, 1.81)	1.14(0.71, 1.85)	0.80(0.49, 1.31)	0.43
Model 1	1.0	1.12(0.69, 1.82)	1.16(0.71, 1.89)	0.83(0.50, 1.37)	0.53
Model 2	1.0	1.11(0.68, 1.80)	1.16(0.71, 1.89)	0.81(0.49, 1.35)	0.51
**Hypertension**					
Crude	1.0	1.78(1.03, 3.09)	2.57(1.50, 4.39)	3.11(1.82, 5.29)	**<0.001**
Model 1	1.0	2.01(1.08, 3.78)	2.03(1.11, 3.71)	2.28(1.25, 4.14)	**0.01**
Model 2	1.0	2.03(1.08, 3.83)	2.12(1.15, 3.09)	2.30(1.26, 4.22)	**0.01**

Abbreviations: LIS: lifestyle inflammation score; HDL, high-density lipoprotein cholesterol; MetS: metabolic syndrome, Q, quartile.

Data are presented as odds ratio (95% CI).

*P-trend is obtained by logistic regression analysis.

Model 1: adjusted for age and energy intake.

Model 2: additionally adjusted for marital status, education status, and occupation.

Supplementary Table 7. Association between the dietary and lifestyle inflammation score and metabolic syndrome in participant who consumed <u><</u>2241 kcal of energy per day (Odd ratios and 95% confidence intervals).

**Variable**	**Q1(n=107)**	**Q2(n=108)**	**Q3(n=102)**	**Q4(n=96)**	**P-trend***
**MetS** (cases, n)	25	40	45	40	
Crude	1.0	1.90(1.05, 3.45)	2.55(1.41, 4.63)	2.31(1.26, 4.23)	**0.004**
Model 1	1.0	1.90(1.03, 3.52)	2.29(1.23, 4.52)	2.06(1.10, 3.85)	**0.02**
Model 2	1.0	1.94(1.04, 3.60)	2.35(1.26, 4.39)	2.06(1.10, 3.87)	**0.02**
**Low HDL**					
Crude	1.0	1.48(0.56, 2.54)	1.17(0.68, 2.03)	0.94(0.53, 1.64)	0.66
Model 1	1.0	1.64(0.91, 2.93)	1.17(0.65, 2.11)	0.90(0.50, 1.64)	0.53
Model 2	1.0	1.69(0.94, 1.03)	1.20(0.66, 1.18)	0.92(0.50, 1.67)	0.56
**Central obesity**					
Crude	1.0	2.09(1.17, 3.72)	3.43(1.92, 6.14)	5.91(3.22, 10.8)	**<0.001**
Model 1	1.0	2.14(1.15, 3.99)	3.10(1.65, 5.83)	6.02(3.10, 11.6)	**<0.001**
Model 2	1.0	2.19(1.17, 4.10)	3.16(1.66, 6.00)	6.63(3.24, 12.4)	**<0.001**
**Hyperglycemia**					
Crude	1.0	0.98(0.57, 1.67)	0.80(0.46, 1.38)	1.05(0.61, 1.38)	0.94
Model 1	1.0	0.98(0.57, 1.68)	0.81(0.47, 1.41)	1.08(0.62, 1.88)	0.97
Model 2	1.0	1.00(0.58, 1.73)	0.82(0.47, 1.44)	1.06(0.60, 1.85)	0.96
**Hypertriglyceridemia**					
Crude	1.0	1.19(0.69, 2.06)	0.95(0.54, 1.67)	0.72(0.40, 1.30)	0.22
Model 1	1.0	1.25(0.72, 2.18)	1.06(0.60, 1.88)	0.80(0.44, 1.14)	0.40
Model 2	1.0	1.24(0.71, 2.18)	1.03(0.57, 1.84)	0.81(0.44, 1.48)	0.41
**Hypertension**					
Crude	1.0	1.83(1.00, 3.32)	2.17(1.20, 3.96)	2.03(1.10, 3.73)	**0.01**
Model 1	1.0	1.80(0.92, 3.52)	1.79(0.91, 3.49)	1.69(0.85, 3.33)	0.16
Model 2	1.0	1.80(0.91, 3.59).	1. 90(0.95, 3.78)	1.66(0.83, 3.34)	0.17

Abbreviations: LIS: lifestyle inflammation score; HDL, high-density lipoprotein cholesterol; MetS: metabolic syndrome, Q, quartile.

Data are presented as odds ratio (95% CI).

*P-trend is obtained by logistic regression analysis.

Model 1: adjusted for age and sex.

Model 2: additionally adjusted for marital status, education status, and occupation**.**

Supplementary Table 8. Association between the dietary and lifestyle inflammation score and metabolic syndrome in participant who consumed ≥2241 kcal of energy per day (Odd ratios and 95% confidence intervals).

Variable	Q1(n=100)	Q2(n=97)	Q3(n=106)	Q4(n=111)	P-trend*
**MetS** (cases, n)	32	29	44	47	
Crude	1.0	0.90(0.49, 1.65)	1.51(0.85, 2.66)	1.56(0.89, 2.43)	**0.04**
Model 1	1.0	0.87(0.46, 1.63)	1.37(0.75, 2.49)	1.37(0.75, 2.49)	0.15
Model 2	1.0	0.83(0.44, 1.58)	1.36(0.74, 2.05)	1.38(0.75, 2.53)	0.14
**Low HDL**					
Crude	1.0	1.05(0.58, 1.88)	1.17(0.66, 2.06)	1.82(1.05, 3.17)	**0.03**
Model 1	1.0	1.04(0.56, 1.94)	1.26(0.68, 2.33)	2.13(1.16, 3.91)	**0.01**
Model 2	1.0	0.99(0.52, 1.86)	1.21(0.65, 2.26)	2.08(1.13, 3.86)	**0.01**
**Central obesity**					
Crude	1.0	1.46(0.83, 2.58)	1.68(0.96, 2.92)	2.12(1.22, 3.67)	**0.007**
Model 1	1.0	1.50(0.80, 2.81)	1.47(0.78, 2.75)	1.88(1.00, 3.53)	0.06
Model 2	1.0	1.48(0.78, 2.83)	1.47(0.78, 2.78)	1.82(0.95, 3.45)	0.08
**Hyperglycemia**					
Crude	1.0	0.98(0.56, 1.72)	0.99(0.57, 1.71)	0.63(0.39, 1.15)	0.17
Model 1	1.0	0.98(0.56, 1.72)	0.99(0.57, 1.73)	0.67(0.39, 1.18)	0.18
Model 2	1.0	1.00(0.56, 1.77)	1.00(0.57, 1.75)	0.71(0.41, 1.25)	0.25
**Hypertriglyceridemia**					
Crude	1.0	0.88(0.49, 1.57)	0.99(0.56, 1.73)	1.29(0.74, 2.23)	0.31
Model 1	1.0	0.87(0.49, 1.56)	0.95(0.54, 1.69)	1.24(0.71, 2.17)	0.39
Model 2	1.0	0.86(0.47, 1.56)	0.96(0.54, 1.71)	1.23(0.70, 2.17)	0.41
**Hypertension**					
Crude	1.0	1.27(0.68, 2.40)	1.97(1.08, 3.58)	2.20(1.22, 3.97)	**0.003**
Model 1	1.0	1.23(0.62, 2.44)	1.59(0.83, 3.04)	1.70(0.90, 3.23)	0.07
Model 2	1.0	1.16(0.58, 2.34)	1.58(0.81, 3.07)	1.71(0.89, 3.29)	0.07

Abbreviations: LIS: lifestyle inflammation score; HDL, high-density lipoprotein cholesterol; MetS: metabolic syndrome, Q, quartile.

Data are presented as odds ratio (95% CI).

*P-trend is obtained by logistic regression analysis.

Model 1: adjusted for age and sex.

Model 2: additionally adjusted for marital status, education status, and occupation.

Supplementary Table 9. Association between the dietary inflammation score and metabolic syndrome in Tehranian adults (Odd ratios and 95% confidence intervals).

**Variable**	**Q1(n=204)**	**Q2(n=207)**	**Q3(n=208)**	**Q4(n=208)**	**P-trend***
**MetS** (cases, n)	72	79	80	75	
Crude	1.0	1.16 (0.78, 174)	1.17 (0.78, 1.74)	1.05 (0.70, 1.58)	0.80
Model 1	1.0	1.21 (0.80, 1.85)	1.18 (0.77, 1.79)	1.07 (0.70, 1.63)	0.98
Model 2	1.0	1.24 (0.81, 1.89)	1.18 (0.77, 1.80)	1.08 (0.71, 1.66)	0.83
**Low HDL**					
Crude	1.0	1.10 (0.74, 1.63)	1.16 (0.78, 1.71)	1.16 (0.78, 1.71)	0.42
Model 1	1.0	1.14(0.75, 1.73)	1.20 (0.79, 1.80)	1.19 (0.78, 1.80)	0.39
Model 2	1.0	1.10(0.72, 1.68)	1.17 (0.77, 1.78)	1.18 (0.78, 1.80)	0.39
**Central obesity**					
Crude	1.0	1.21(0.82, 1.78)	1.13(0.77, 1.66)	0.95(0.64, 1.40)	0.73
Model 1	1.0	1.31(0.85, 2.02)	1.15(0.75, 1.77)	0.96(0.62, 1.47)	0.71
Model 2	1.0	1.33(0.86, 2.06)	1.15(0.75, 1.77)	0.98(0.64, 1.52)	0.79
**Hyperglycemia**					
Crude	1.0	0.92(0.63, 1.36)	0.80(0.54, 1.17)	0.75(0.51, 1.11)	0.11
Model 1	1.0	0.92(0.63, 1.36)	0.80(0.54, 1.17)	0.75(0.51, 1.11)	0.11
Model 2	1.0	0.95(0.64, 1.41)	0.81(0.55, 1.19)	0.75(0.51, 1.11)	0.10
**Hypertriglyceridemia**					
Crude	1.0	1.22(0.82, 1.81)	0.95(0.63, 1.42)	1.07(0.72, 1.60)	0.96
Model 1	1.0	1.21(0.81, 1.80)	0.96(0.64, 1.43)	1.07(0.72, 1.60)	0.98
Model 2	1.0	1.22(0.81, 1.81)	0.95(0.63, 1.42)	1.08(0.73, 1.61)	0.99
**Hypertension**					
Crude	1.0	0.92(0.60, 1.39)	1.35(0.90, 2.02)	1.12(0.75, 1.69)	0.21
Model 1	1.0	0.93(0.59, 1.48)	1.38(0.89, 2.16)	1.19(0.76, 1.87)	0.19
Model 2	1.0	0.93(0.58, 1.48)	1.39(0.89, 2.19)	1.20(0.76, 1.89)	0.19

Abbreviations: DLIS: dietary and lifestyle inflammation score; HDL, high-density lipoprotein cholesterol; MetS: metabolic syndrome, Q, quartile.

Data are presented as odds ratio (95% CI).

*P-trend is obtained by logistic regression analysis.

Model 1: adjusted for age, sex, and energy intake.

Model 2: additionally adjusted for marital status, education status, and occupation**.**

Supplementary Table 10. Association between the lifestyle inflammation score and metabolic syndrome in Tehranian adults (Odd ratios and 95% confidence intervals).

**Variable**	**Q1(n=222)**	**Q2(n=141)**	**Q3(n=256)**	**Q4(n=204)**	**P-trend***
**MetS** (cases, n)	55	40	116	95	
Crude	1.0	1.19(0.74, 1.92)	2.18(1.48, 3.22)	2.63(1.74, 3.96)	**<0.001**
Model 1	1.0	1.05(0.64, 1.73)	1.87(1.24, 2.82)	2.36(1.51, 3.68)	**<0.001**
Model 2	1.0	1.07(0.64, 1.77)	1.92(1.27, 2.92)	2.42(1.54, 3.81)	**<0.001**
**Low HDL**					
Crude	1.0	0.91(0.59, 1.39)	0.95(0.67, 1.37)	0.76(0.52, 1.13)	0.93
Model 1	1.0	1.00(0.63, 1.60)	1.02(0.69, 1.52)	0.91(0.59, 1.41)	0.99
Model 2	1.0	0.97(0.61, 1.56)	1.00(0.67, 1.49)	0.90(0.58, 1.41)	0.97
**Central obesity**					
Crude	1.0	3.98(2.49, 6.37)	5.33(3.54, 8.02)	9.75(6.23, 15.2)	**<0.001**
Model 1	1.0	3.74(2.25, 6.22)	4.78(3.06, 7.47)	10.6 (6.40, 17.7)	**<0.001**
Model 2	1.0	3.61(2.16, 6.05)	4.78(3.04, 7.52)	10.2(6.12, 17.1)	**<0.001**
**Hyperglycemia**					
Crude	1.0	0.43(0.28, 0.64)	1.10(0.77, 1.57)	1.40(0.95, 2.05)	**0.009**
Model 1	1.0	0.45(0.29, 0.72)	1.16(0.80, 1.68)	1.49(0.99, 2.22)	**0.003**
Model 2	1.0	0.47(0.29, 0.74)	1.19(0.82, 1.72)	1.52(1.01, 2.29)	**0.002**
**Hypertriglyceridemia**					
Crude	1.0	0.62(0.39, 0.96)	0.86(0.60, 1.24)	0.85(0.58, 1.26)	0.67
Model 1	1.0	0.64(0.40, 1.01)	0.90(0.62, 1.31)	0.89(0.59, 1.34)	0.90
Model 2	1.0	0.62(0.39, 0.98)	0.89(0.61, 1.30)	0.87(0.58, 1.32)	0.86
**Hypertension**					
Crude	1.0	2.42(1.51, 3.89)	2.32(1.54, 3.50)	3.08(2.01, 4.73)	**<0.001**
Model 1	1.0	1.66(0.95, 2.81)	1.45(0.92, 2.29)	1.71(1.06, 2.76)	**0.05**
Model 2	1.0	1.77(1.04, 3.03)	1.53(0.96, 2.44)	1.85(1.13, 3.01)	**0.03**

Abbreviations: LIS: lifestyle inflammation score; HDL, high-density lipoprotein cholesterol; MetS: metabolic syndrome, Q, quartile.

Data are presented as odds ratio (95% CI).

*P-trend is obtained by logistic regression analysis.

Model 1: adjusted for age, sex, and energy intake.

Model 2: additionally adjusted for marital status, education status, and occupation.

Supplementary Table 11. Association between the lifestyle inflammation score (without body mass index) and metabolic syndrome in Tehranian adults (Odd ratios and 95% confidence intervals).

**Variable**	**Q1(n=291)**	**Q2(n=456)**	**Q3(n=80)**	**P-trend***
**MetS** (cases, n)	84	199	27	
Crude	1.0	1.74(1.27, 2.38)	1.24(0.73, 2.11)	**0.005**
Model 1	1.0	1.76(1.27, 2.44)	1.68(0.94, 2.99)	**0.001**
Model 2	1.0	1.73(1.25, 2.04)	1.66(0.92, 2.96)	**0.001**
**Low HDL**				
Crude	1.0	0.81(0.60, 1.08)	0.60(0.35, 1.00)	**0.05**
Model 1	1.0	0.73(0.53, 1.01)	1.02(0.57, 1.82)	0.17
Model 2	1.0	0.73(0.53, 1.00)	1.04(0.58, 1.86)	0.17
**Central obesity**				
Crude	1.0	1.28(0.96, 1.72)	1.17(0.71, 1.93)	0.13
Model 1	1.0	1.30(0.94, 1.80)	1.85(1.03, 3.33)	**0.03**
Model 2	1.0	1.29(0.93, 1.80)	1.90(1.05, 3.41)	**0.03**
**Hyperglycemia**				
Crude	1.0	2.83(2.09, 3.84)	2.32(1.40, 3.84)	**<0.001**
Model 1	1.0	2.83(2.09, 3.84)	3.36(1.41, 3.95)	**<0.001**
Model 2	1.0	2.83(2.09, 3.84)	2.29(1.37, 3.84)	**<0.001**
**Hypertriglyceridemia**				
Crude	1.0	1.57(1.16, 2.14)	1.32(0.78, 2.21)	**0.01**
Model 1	1.0	1.60(1.18, 2.18)	1.33(0.78, 2.25)	**0.009**
Model 2	1.0	1.60(1.17, 2.17)	1.34(0.78, 2.27)	**0.01**
**Hypertension**				
Crude	1.0	0.83(0.61, 1.12)	1.01(0.60, 1.68)	0.42
Model 1	1.0	0.80(0.57, 1.13)	0.82(0.45, 1.47)	0.22
Model 2	1.0	0.77(0.55, 1.09)	0.78(0.43, 1.40)	0.15

Abbreviations: LIS: lifestyle inflammation score; HDL, high-density lipoprotein cholesterol; MetS: metabolic syndrome, Q, quartile.

Data are presented as odds ratio (95% CI).

*P-trend is obtained by logistic regression analysis.

Model 1: adjusted for age, sex, and energy intake.

Model 2: additionally adjusted for marital status, education status, and occupation.

Supplementary Table 12. Modified dietary and lifestyle inflammation score components.

Components	Rationales for inclusion	Weights
	**DIS components**	
**Leafy greens and cruciferous vegetables**	Kale, spinach, lettuce (iceberg, head, romaine, or leaf), broccoli, Brussels sprouts, cabbage, cauliflower, parsley, watercress	-0.14
**Tomatoes**	Tomatoes, tomato juice, tomato sauce	-0.78
**Apples and berries**	Fresh apples, pears, apple juice or cider, strawberries, blueberries, raspberries, cherries	-0.65
**Deep yellow or orange vegetables and fruit**	Cantaloupe, peaches, carrots, dark yellow or orange squash, figs	-0.57
**Other fruits and real fruit juices**	Fresh fruits other than those listed above (e.g. pineapples, honeydew, grapes, kiwi, watermelon, lemon, grapefruit, and oranges), orange juice, grapefruit juice, grape juice, and other real fruit juice	-0.16
**Other vegetables**	Vegetables other than those listed above (e.g., okra, green peppers, onions, zucchini, and eggplant)	-0.16
**Legumes**	String beans, peas, lentils, and other beans (excluding soybeans)	-0.04
**Fish**	Tuna fish, salmon, other light and dark meat fish, breaded fish cakes or fish sticks	-0.08
**Poultry**	Chicken or turkey with and without skin	-0.45
**Red and organ meats**	Hamburger, beef, lamb, liver, gizzards, other organ meats	0.02
**Processed meats**	Bacon, beef hotdogs, chicken or turkey hot dogs, other processed meats	0.68
**Added sugars**	Sugar-sweetened soda, punch, lemonade, chocolate candy bars, other mixed candy bars, candy without chocolate, jams, jellies, preserves, syrup or honey, dried or canned fruit	0.56
**High-fat dairy**	Whole milk, 2% milk, cream, high-fat ice cream, high-fat yogurt, cream cheese, other high-fat cheeses	-0.14
**Low-fat dairy**	Skim milk, 1% milk, low-fat yogurt, low-fat ice cream, low-fat cheeses	-0.12
**Coffee and tea**	Coffee (decaffeinated and regular), herbal and non-herbal tea	-0.25
**Nuts**	peanuts, other nuts	-0.44
**Other fats**	Mayonnaise, margarine, butter, vegetable oil	0.31
**Refined grains and starchy**	Cold and cooked breakfast cereal, white or dark bread, bagels, rolls, corn	0.72

**vegetables**	bread, white rice, pasta, pancakes, waffles, potatoes (French fried, scalloped, baked, boiled or mashed), sweet potato/yams, potato chips, crackers, tortillas, popcorn, pretzels, cookies, brownies, doughnuts, cake, pie, sweet rolls, coffee cakes	
**LIS components**		
**Moderately physically active**	600-3000 MET/minute/week	-0.18
**Heavily physically active**	>3000 MET/minute/week	-0.41
**Current smoker**	Currently smokes vs. does not currently smokers	0.50
**Overweight BMI**	Overweight BMI vs. normal BMI	0.89
**Obese BMI**	Obese BMI vs. normal BMI	1.57

Abbreviations: BMI, body mass index; DIS, dietary inflammation score; LIS, lifestyle inflammation score
